# Molecular epidemiology of rabies virus in Poland

**DOI:** 10.1007/s00705-014-2045-z

**Published:** 2014-03-14

**Authors:** Anna Orłowska, Jan Franciszek Żmudziński

**Affiliations:** Department of Virology, National Veterinary Research Institute, Partyzantów 57 Avenue, 24-100 Puławy, Poland

## Abstract

The paper describes a phylogenetic study of 58 Polish isolates of rabies virus collected between 1992 and 2010. Sequences of the nucleoprotein (N) and glycoprotein (G) genes approximately 600 bp long were compared with reference sequences (GenBank) of European rabies viruses from neighbouring countries. The study confirmed a very high level of homology (94.4–100 %) of the Polish rabies virus strains irrespective of the date of isolation. Two variants of rabies virus: NEE (Northeastern Europe variant) and CE (Central Europe variant), depending on the geographical place of isolation, were circulating in Poland from 1992 to 2010. The Polish rabies virus isolates showed high similarity to European RABV strains, especially those collected in Ukraine and Romania. They were clearly different from vaccine strains SAD B19 and SAD Bern, which have been used for oral vaccination of foxes against rabies in Poland since 1993.

## Introduction

Rabies is an acute viral infection of the central nervous system caused by rabies virus, a member of the order *Mononegavirales*, family *Rhabdoviridae* and genus *Lyssavirus*. Among the lyssaviruses, based on the sequence analysis and phylogenetics, 12 species have been recognized, which are sometimes also referred as genotypes [4].

Rabies is recorded in many countries throughout the world, with the exception of some islands, archipelagos and countries. In Poland, the main reservoir of rabies virus (RABV) is red fox (*Vulpes vulpes*) [9, 12, 22, 30]. To reduce the number of rabies cases in Poland, oral rabies vaccination (ORV) of foxes was introduced in 1993. At the beginning, vaccination was conducted in western Poland, along the Polish–German border. Since 2002, the vaccine has been distributed over the whole territory of Poland. Two vaccines, Fuchsoral and Lyssvulpen, containing attenuated live rabies virus strains SAD B19 and SAD Bern, respectively, are distributed twice a year. Despite the wide distribution of oral vaccine, rabies cases are still diagnosed in Poland, especially in the southeastern part of the country neighbouring with Ukraine, Belarus and Lithuania. Due to the fact that oral rabies vaccines contain live, attenuated rabies virus strains with the potential for replication and reversion to the pathogenic form, all field isolates have to be tested in differential tests against vaccine strains. Rabies cases in red foxes associated with the vaccine have been diagnosed in Germany and Austria [19].

Nucleoprotein is the most conserved of the viral components in terms of amino acid sequence similarity within species. Thus, the nucleoprotein plays a crucial role in rabies virus detection. Also, evolutionary studies of lyssaviruses have tended to focus on the N protein. Four phylogenetic groups have been distinguished in Europe since 1999 based on comparisons of nucleotide sequences of the N gene [3]. The other protein of rabies virus, glycoprotein (G), is a surface viral protein containing domains responsible for host-cell receptor recognition [27] and membrane fusion [8] and is a major target for the host neutralizing-antibody response [1]. However, some evolutionary studies based on phylogenetic analysis of nucleotide sequences of the G gene have also been conducted [13].

Molecular study allows for better analysis of rabies epidemiology, and thus a genetic characterization of the Polish RABV strains in relation to reference rabies virus sequences from other European countries available in the GenBank database has been carried out. The sequences of the N and G genes of the 58 Polish field isolates collected between 1992 and 2010 were analysed. A previous study included field strains collected between 1985 and 1996 [3]. It has been shown that Polish rabies virus isolates can be classified into four phylogenetic groups distinguished in Europe since 1999 based on the comparison of nucleotide sequences of the N gene [3].

## Materials and methods

### Samples

The study included 58 Polish RABV isolates collected in Poland between 1992 and 2010. The samples were obtained from regional veterinary laboratories and were diagnosed as rabies positive by fluorescent antibody test (FAT) [6] with anti-nucleocapsid conjugate (Bio-Rad). Positive samples originated mainly from foxes. Single samples were obtained from raccoon dogs, cattle, dogs, cats and a polecat (Table 1). As negative controls of RT-PCR brains of mice were taken. To prove that the rabies outbreaks were not caused by the vaccine strains, molecular comparison of analyzed RABV nucleotide sequences was also done against vaccine SAD strains (SAD B19 and SAD Bern).

**Table 1:** Description of the origin of Polish rabies virus isolates used in present study

Strain designation^a^	Collection date	Region	Species	GenBank accession number (N)
A/1993/K/POL	1993	Zachodnio-Pomorskie	Cat	JN596880
A/1993/L/POL	1993	Zachodnio-Pomorskie	Fox	JN190357
A/1994/L/POL	1994	Zachodnio-Pomorskie	Fox	JN190358
C/1994/L/POL	1994	Dolnoslaski	Fox	JN190359
D/1994/B/POL	1994	Opolskie	Cattle	JN190361
D/1992/L/POL	1992	Opolskie	Fox	JN190362
E/1992/K/POL	1992	Wielkopolskie	Cat	JN190385
R/1996/L/POL	1996	Slaskie	Fox	JN190364
O/1994/L/POL	1994	Podkarpackie	Fox	JN190365
O/1994/Tch/POL	1994	Podkarpackie	Polecat	JN596881
F/1994/B/POL	1994	Kujawsko-Pomorskie	Cattle	JN596882
L/1994/L/POL	1994	Lodzkie	Fox	JN190367
L/1995/L/POL	1995	Lodzkie	Fox	JN596883
K/1994/L/POL	1994	Mazowieckie	Fox	JN190368
B/1995/L/POL	1995	Lubuskie	Fox	JN190369
H/1996/J/POL	1996	Warmiansko-Mazurskie	Raccoon dog	JN596884
P/1996/B/POL	1996	Małopolskie	Cattle	JN596885
P/1996/P/POL	1996	Małopolskie	Dog	JN190371
M/1996/L/POL	1996	Swietokrzyskie	Fox	JN190372
M/1996/P/POL	1996	Swietokrzyskie	Dog	JN596886
N/1996/L/POL	1996	Lublskie	Fox	JN596887
N/1996/L/1/POL	1996	Lublskie	Fox	JN596888
O/1996/L/POL	1996	Podkarpackie	Fox	JN596889
L/2000/L/POL	2000	Lodzkie	Fox	JN190373
L/2000/L/1/POL	2000	Lodzkie	Fox	JN596890
D/2001/L/POL	2001	Opolskie	Fox	JN190374
C/2002/L/POL	2002	Dolnoslaskie	Fox	JN190375
E/2002/L/POL	2002	Wielkopolskie	Fox	JN596891
E/2002/P/POL	2002	Wielkopolskie	Fox	JN596892
A/2004/L/POL	2004	Zachodnio-Pomorskie	Fox	JN596893
F/2001/L/POL	2001	Zachodnio-Pomorskie	Fox	JN190376
F/2001/P/POL	2001	Kujawsko-Pomorskie	Dog	JN596894
H/2000/L/POL	2000	Warminsko-Mazurskie	Fox	JN190377
H/2000/J/POL	2000	Warminsko-Mazurskie	Raccoon dog	JN596895
P/2000/L/POL	2000	Maloposkie	Fox	JN190378
B/2003/L/POL	2003	Lubuskie	Fox	JN190379
B/2003/K/POL	2003	Lubuskie	Cat	JN596896
K/2000/Br/POL	2000	Mazowieckie	Badger	JN596897
M/2001/L/POL	2001	Swietokrzyskie	Fox	JN596898
M/2001/L/1/POL	2001	Swietokrzyskie	Fox	JN190381
N/2001/L/POL	2001	Lubelskie	Fox	JN190382
N/2001/L/1/POL	2001	Lubelskie	Fox	JN596899
J/2003/B/POL	2003	Podlasklie	Cattle	JN596900
J/2003/L/POL	2003	Podlaskie	Fox	JN190383
E/2003/L/POL	2003	Wielkopolskie	Fox	JN190386
E/2003/K/POL	2003	Wielkopolskie	Cat	JN190387
E/2004/K/POL	2004	Wielkopolskie	Cat	JN596901
E/2005/L/POL	2005	Wielkopolskie	Fox	JN596902
E/2005/P/POL	2005	Wielkopolskie	Dog	JN596903
E/2006/L/POL	2006	Wielkopolskie	Fox	JN596904
E/2006/P/POL	2006	Wielkopolskie	Dog	JN596905
E/2007/L/POL	2007	Wielkopolskie	Fox	JN190389
H/2008/J/POL	2008	Warminsko-Mazurskie	Raccoon dog	JN190392
H/2008/B/POL	2008	Warminsko-Mazurskie	Cattle	JN190391
O/2009/L/POL	2009	Podkarpackie	Fox	JN190393
N/2009/L/POL	2009	Lubelskie	Fox	JN596910
P/2010/L/POL	2010	Malopolskie	Fox	JN190395
P/2010/B/POL	2010	Malopolskie	Cattle	JN596912

^a^The strain names include the year of isolation, the host species, and “POL” for Poland

### RNA extraction

A sample of brain tissue was homogenized in sterile water for injection, and total RNA was extracted using a commercial kit (QIAmp Viral RNA Mini Kit, QIAGEN) according to the manufacturer’s instructions. Pellets were resuspended in RNAse-free water in a final volume of 50 μl and used immediately for RT-PCR. The remaining RNA was stored frozen at −20 °C.

### RT-PCR

Reverse transcription and polymerase chain reaction were performed using previously published methods [20, 24] to amplify a 600-bp region of the nucleoprotein gene of RABV. The primers JW12 (5′-ATG TAA CAC CYC TAC AAT G-3′) and JW6DPL (5′-CAA TTC GCA CAC ATT TTG TG-3′) were published by Heaton et al. [10].

To amplify a 590-bp fragment of the G gene of RABV, the primer set Gp2L (5′-AGT AGA GGG AAG AGA GCA TCC A-3′) and Gp2P (5′-GAG GAT AGG AAC AAC TCC AT-3′), corresponding to nt 3957–4547 of the PV reference strain (accession no. M13215) of RABV was designed based on the alignment of sequences of different rabies virus isolates published in GenBank. The RT-PCR assay was carried out using a OneStep RT-PCR Kit (QIAGEN). Briefly, two microlitres of total RNA was added to a mixture containing 3 μl of 5× OneStep RT-PCR buffer, 0.4 μl of each dNTP at a concentration of 10 mM, 0.5 μl of RNAse inhibitor, 0.6 μl of enzyme mix, 1 μl of sense and antisense primers at a concentration of 10 mM, and 6.5 μl of RNAse-free water to make a final volume of 15 μl. Amplification was performed in a Personal Cycler (Biometra) using the following program: one cycle of RT at 50 °C for 30 min, followed by denaturation at 95 °C for 15 min, 35 cycles with denaturation at 95 °C for 30 s, annealing at 58 °C for 30 s, and elongation at 72 °C for 1 min, and a final extension at 72 °C for 10 min.

Amplified products (amplicons) were visualized by agarose gel electrophoresis and were purified using a commercial kit (QIAquick PCR Purification Kit, QIAGEN). Purified amplicons were sequenced in both directions using an automated sequencer (ABI PRISM 310 Genetic Analyzer, Applied Biosystems) using a BigDye Sequencing Kit (Applied Biosystems) with GeneScan Analysis Software, using the same primers as used for RT-PCR.

### Phylogenetic analysis

Nucleotide sequences of antisense strands after sequencing were reversed using the Reverse Complement program. Fifty-eight nucleotide sequences from analyzed RABV isolates were aligned using Clustal W multiple alignment and visualized with the BioEdit software v. 7.0.5.3. Multiple sequence alignments were done based on the 570-bp regions of each nucleoprotein and glycoprotein gene. The similarity matrix was made using BLOSUM62 in the BioEdit program. A phylogenetic tree was generated using the neighbour-joining (NJ) method with the Kimura 2-parameter model and 1000 bootstrap replicates with the Mega software v. 4.1 [25]. To determine the phylogenetic relationship of Polish RABVs, 58 N and G nucleotide sequences were compared to reference sequences (available in the GenBank database), taking into consideration close relationships and geographic criteria (Table 2). As vaccine reference strains, sequences of PV, SAD B19 (accession no. EF206709) and SAD Bern (accession no. EF206708) were used for analysis.

**Table 2:** Characteristics of rabies virus isolates included in phylogenetic study

Country	Isolate	Collection date	Species	GenBank accession number (N)	GenBank accession number (G)	References
Germany	9202ALL	1991	Red fox	U42701	AF134338	Bourhy et al. [3]
	9212ALL	1991	Red fox	U22475	AF134337	
	9213ALL			U42702		Bourhy et al. unpublished
	Rv313	1990	Red fox		GU936875	Horton et al. unpublished
Poland	8618POL	1985	Raccoon dog	U22840		Kissi et al. [14]
France	9353FRA	1993	Red fox		AF134327	Bourhy et al. [3]
	9445FRA	1994	Red fox	U42700	AF134332	
Hungary	9384HON	1993	Red fox		AF134340	Bourhy et al. [3]
	9386HON			U43000		Bourhy et al. unpublished
	9383HON			U42998		
Slovenia	664-02SVN	2002	Fox		HM852168	Rihtaric et al. [23]
Bosnia and Herzegovina	8653YOU	1986	Wolf	U42704	AF134341	Bourhy et al. [3]
Former Yugoslavia	86106YOU	1972	Red fox	U22839		Kissi et al. [14]
Bulgaria	Bul#7	2003	Fox	DQ300294		Johnson et al. [11]
Estonia	9339EST	1991	Raccoon dog	U42707	AF134335	Bourhy et al. [3]
	9142EST	1985	Raccoon dog	U22476	AF134339	
Ukraine	Rvu10-04	2010	Dog	JN656503		Picard-Meyer et al. [21]
	Rvu10-09	2010	Fox		JN656522	
	Rvu10-14	2010	Stray dog		JN656506	
	Rvu02-16	2002	Fox	JN656502		
Romania	RO-RV-2537-06-VL	2006	Red fox	GU086619		Turcitu et al. [26]
	RO-RV-2534-06-SM	2006	Red fox	GU086614		
Lithuania	06LT4		Raccoon dog	EU616717		Zienius et al. [31]

## Results

The presence of viral RNA (the N and G fragments of genes) was detected in all 58 samples that had been diagnosed previously as positive by FAT. RT-PCR products of the expected size of 600 bp for the N gene and 590 bp for G were also obtained for the positive control (PV strain propagated in cell culture). No amplification was observed in the negative controls.

Comparison of N and G gene nucleotide sequences of Polish RABV isolates with those of the reference strains PV and SAD B19/SAD Bern showed that the homology ranged from 90.9–92.7 % and 91.1–93.2 % for the PV and SAD strain, respectively. The identity of deduced amino acid sequences of nucleoprotein and glycoprotein (191 aa of each) was higher than the identity of nucleotide sequences and ranged from 93.7 to 95.8 (PV strain) and from 94.7 to 96.8 % (SAD B19 and SAD Bern strains). Many of the nucleotide substitutions in the analyzed fragments of the N and G genes of Polish RABV isolates were synonymous, and thus the amino acid sequences of the proteins were not modified.

Phylogenetic analysis based on a dataset of nucleoprotein nucleotide sequences of the Polish RABV isolates and reference strains (PV, SAD Bern and SAD B19) produced a tree with two branches. The first consisted of the Polish RABV isolates, and the second one contained the PV and SAD strains (data not shown).

The polish RABV isolates showed high homology, ranging from 94.4 to 100 %, based on the analysis of N and G gene nucleotide sequences. Almost 99.8 % homology was observed among Polish RABV isolates collected in the 1990s and in 2010. The identity of deduced amino acid sequences of nucleoprotein and glycoprotein (191 aa of each) ranged from 95.8 to 100 %.

**Fig. 1:**
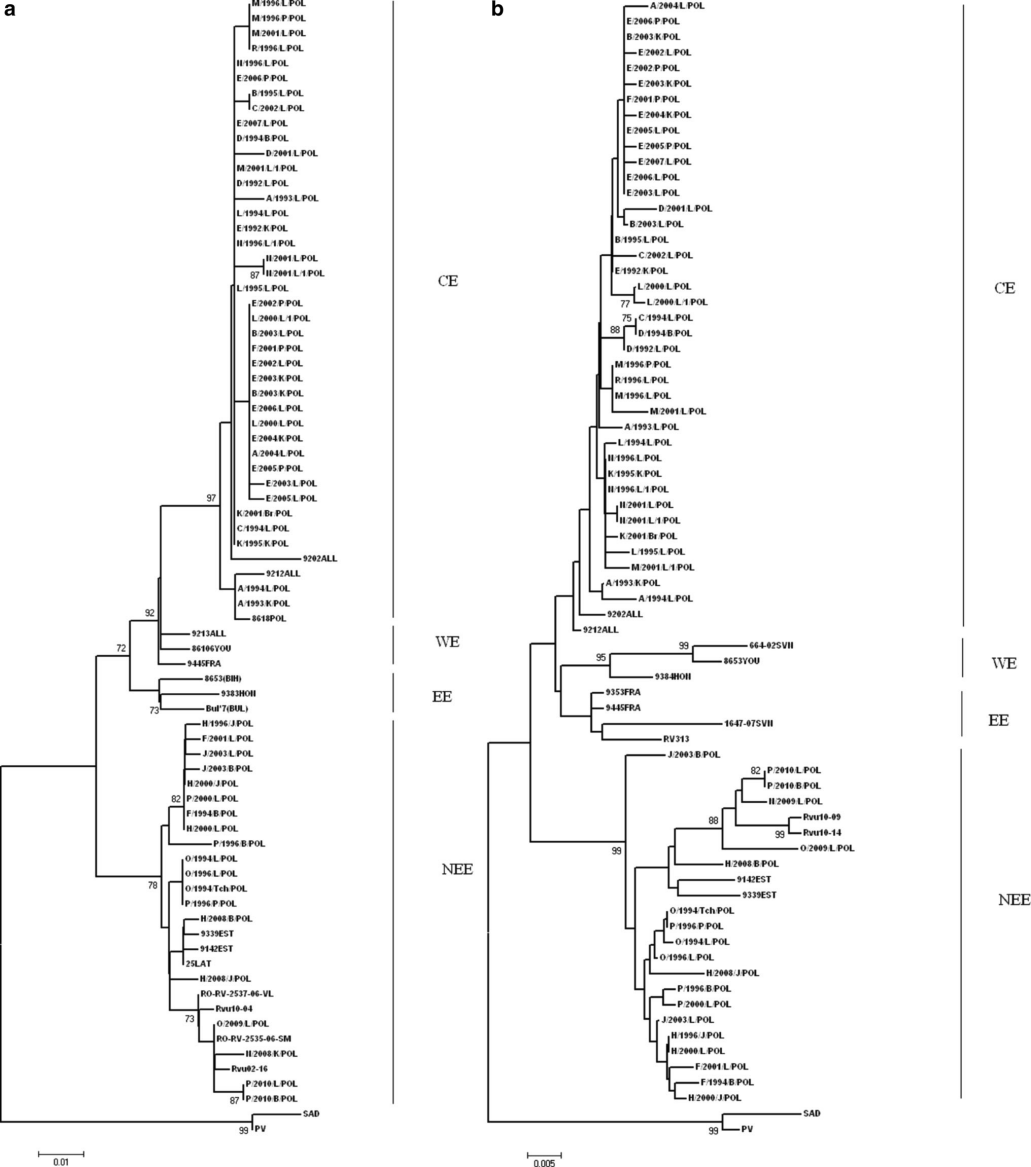
Phylogenetic trees comparing the Polish RABV isolates with the reference European rabies virus strains based on N (a) and G (b) gene nucleotide sequences. The phylogenetic analysis was conducted by the NJ method. Bootstrap values were obtained for 1000 replicates. “Vaccine SAD strain” represents both the SAD B19 and the SAD Bern strains. PV and SAD strains were used as outgroup

Two phylogenetic groups, NEE and CE, were reported in Poland from 1992 to 2010. Phylogenetic analysis of the dataset (n = 75) of N gene sequences using the NJ method is shown in Fig. 1a. Thirty-nine sequences of Polish RABV strains belonged to the CE group, together with two nucleotide sequences from Germany. The remaining 19 N gene sequences formed the NEE group, together with East European rabies virus strains. The closest relationship was observed between Polish RABV strains and Ukrainian and Romanian strains, supported by a bootstrap value 73.

## Discussion

The principal objective of this study was to determine the phylogenetic relationships between Polish RABV field strains and European rabies virus variants, especially those originating from neighboring countries. To prove that the rabies outbreaks between 1992-2010 were not caused by the vaccine strains used in ORV, a molecular comparison of analyzed RABV nucleotide sequences was done with vaccine SAD strains (SAD Bern and SAD B19). A phylogenetic tree constructed based on the nucleotide sequences of nucleoprotein gene of Polish RABV isolates and reference strains (PV, SAD Bern and SAD B19) showed that rabies vaccine SAD strains used in ORV in Poland did not contribute to rabies outbreaks between 1992 and 2010. Moreover, a comparison of nucleotide as well as amino acid sequences of nucleoproteins and glycoproteins of the Polish RABV strains and SAD strains (data not shown) confirmed that no rabies cases had been caused by a vaccine strain, which may also indicate the genetic stability of the attenuated vaccine strains distributed in Poland for ORV and that they do not revert to a pathogenic form. Although live attenuated oral rabies vaccines could revert to virulence and have low residual pathogenicity in certain rodents, it has been shown that they are safe for foxes, dogs and skunks [28, 29]. Intensive molecular investigation of SAD B19 strains passaged several times in mice has demonstrated their sequence conservation and genetic stability *in vivo* [2]. However, it should be considered that there is always a risk that the vaccine strain may revert to a fully pathogenic form, and thus, all rabies virus isolates collected from the field should be sequenced and subjected to phylogenetic analysis. In Poland, since 2007, all rabies field isolates have been sequenced and analyzed.

The high homology observed among Polish RABV isolates collected in the 1990s and in 2010 suggests the circulation of the same strain of RABV in the field for almost 20 years. Moreover, phylogenetic analysis revealed the high similarity of 1992-2010 isolates to the Polish strain (8618 POL) isolated in 1985, the sequence of which is available in GenBank. Polish RABV isolates collected at the end of the 20th century have shown high similarity to the field RABV strains from Germany, Estonia, and the other republics of the former Soviet Union, whereas RABV isolates collected in 2008-2010 have shown the highest homology with Ukrainian and Romanian strains of rabies virus. Recently, rabies virus in terrestrial animals in Poland has been detected mainly in the provinces located in the eastern and southeastern part of the country, and thus the homology to Ukrainian and Romanian strains is not surprising. These results are very important for epidemiological study. It is very likely that Polish RABV strains collected in northeastern Poland are closely related to rabies virus isolates circulating in the Kaliningrad region. Chupin et al. [5] presented data on the classification of RABV variants in Russia based on the analysis of a 334-bp-long N gene fragment. The analysis of 63 isolates revealed that 15 variants belonged to the Eurasiatic group, with a variation of 0-3.9 %. Thirty-six isolates belonged to the Central group, showing a close relationship to European variants of RABV. Variant RV262 of Briansk was closely related (97.3 %) to rabies virus isolates from Hungary (9215HON). Four isolates were closely related to the North European group of RABV, and all of the isolates from the northwestern part of Russia were related to the North European RABV group. However, the comparison of the Russian and Polish isolates may not have been precise, as various fragments of the N gene were analyzed. For Polish isolates, nucleotides 55–660 in the N gene were examined, and in case of the Russian isolates it was 582–915. Metlin et al. [17, 18] have found an arctic RABV strain circulating in the European part of Russia (Pskov, Kursk, Tver). None of the Polish isolates were related to this strain.

The high homology of Polish RABV strains to Ukrainian and Romanian RABV strains seems to be connected with the epizootic status of rabies in these countries. Keeping in mind that about 2000 cases of rabies occur annually in Ukraine as well as in other republics of the former Soviet Union and in the Balkans (source: http://www.rbe.fli.bund.de/), migration of rabid wildlife to Poland from neighbouring countries is highly probable. As has been suggested by Picard-Meyer et al. [21], it is very likely that some or all of the cases of rabies in the Polish provinces Podkarpackie and Lubelskie are due to migration of rabid animals from Ukraine to Poland. In this study, we found more than 99.1 % nucleotide sequence identity in RABV isolates from Poland, Ukraine and Romania. The persistence of rabies in animals along the borders is a permanent threat because of migration of rabid animals. Johnson et al. [11] demonstrated the migration of vectors of rabies virus between Balkan states. The rabies outbreak in 2008 in Italy also appeared in an area bordering with Slovenia, and it expanded through the northwestern provinces [7]. In the central and southwestern part of Poland neighbouring with Germany and the Czech Republic, the rabies status is not influenced by neighbours due to the fact that those countries are rabies free or have only sporadic cases of rabies [15, 22].

Polish RABV isolates formed two distinct groups of closely related strains belonging to the Northeastern European (NEE) and Central European (CE) groups as described by Bourhy et al. [3]. The clustering of RV variants by geographical region showed that the NEE group is mainly observed in the eastern part of Poland, while all CE group isolates except four from Lubelskie, Podlaskie and Podkarpackie provinces were limited to the Polish territory on the western bank of the Vistula River. These results correspond to the geographical distribution of rabies variants in Europe. The NEE group is found in western Russia, Finland, Ukraine, Estonia, Lithuania, Latvia, Romania and Slovakia [3, 16, 21, 26, 31], while the CE group is found in eastern Germany, the Czech Republic, and in Slovenia [3, 16]. The Vistula River, which divides the territory of Poland into two parts, might be a natural border in the distribution of RV variants (NEE and CE), as was previously suggested by Bourhy et al. [3]. However, during the winter, when the river is frozen, rabid animals could cross the river. Animals can also by the bridge, and therefore, CE rabies viruses have been identified in Polish provinces located on the right bank of the Vistula River. No rabies isolates belonging to the EE and WE groups were identified in the current study despite the fact that they were described previously (two isolates of the EE group were identified between 1992 and 1994, and two isolates of the WE group were identified in 1995 [3]). Although our study includes isolates collected from the whole territory of Poland, it is possible that neither EE nor WE isolates were detected over the time of the study. Members of the NEE and CE groups are the most frequently rabies viruses in Poland. Only single cases of WE and EE have been diagnosed previously in Poland, and thus it is possible that they are not currently circulating in Poland.

In conclusion, two variants of rabies virus circulating in Poland between 1992 and 2010 were identified, and their sequences were compared to those of other rabies viruses available in the GenBank database. This study confirmed the very high level of homology of all Polish rabies virus strains, irrespective of the time of isolation. The Polish RABV isolates show a close relationship to other European RABV strains. RABV strains collected from 2008 to 2010 showed high homology to Ukrainian and Romanian isolates, whereas Polish RABV strains isolated at the end of the 20th century were similar to German strains.

## References

[1] Badrane H, Bahloul C, Perrin P, Tordo N (2001). Evidence of two *Lyssavirus* phylogroups with distinct pathogenicity and immunogenicity. J Virol.

[2] Beckert A, Geue L, Vos A, Neubert A, Freuling C, Müller T (2009). Genetic stability (in vitro) of the attenuated oral rabies virus vaccine SAD B19. Microbiol Immunol.

[3] Bourhy H, Kissi B, Audry L, Smreczak M, Sadkowska-Todys M, Kulonen K, Tordo N, Zmudzinski JF, Holmes E (1999). Ecology and evolution of rabies virus in Europe. J Gen Virol.

[4] Carstens EB (2010). Ratification vote on taxonomic proposals to the International Committee on Taxonomy of Viruses (2009). Arch Virol.

[5] Chupin SA, chernyshova EV, Metlin AE (2013). Genetic characterization of the rabies virus field isolates detected in Russian Federation within the period 2008–2011. Vopr Virusol.

[6] Dean DJ, Abelseth MK, Atanasiu P, Meslin FX, Kaplan MM, Koprowski H (1996). The fluorescent antibody test. Laboratory techniques in rabies.

[7] De Benedictis P, Gallo T, Iob A, Coassin R, Squecco G, Ferri G, Ancona FD, Marangon S, Capua I, Mutinelli F (2008). Emergence of fox rabies in North-Eastern Italy. Euro Surrveill.

[8] Durrer P, Gaudin Y, Ruigrok RW, Graf R, Brunner J (1995). Photolabeling identifies a putative fusion domain in the envelope glycoprotein of rabies and vesicular stomatitis viruses. J Biol Chem.

[9] Finnegan CJ, Brookes SM, Johnson N, Smith J, Mansfield KL, Keene VL, McElhinney LM, Fooks AR (2002). Rabies in North America and Europe. J R Soc Med.

[10] Heaton PR, Johnstone P, McElhinney LM, Cowley R, O’Sullivan E, Whitby JE (1997). Heminested PCR assay for detection of six genotypes of rabies and rabies-related viruses. J Clin Microbiol.

[11] Johnson N, Fooks AR, Valtchovski R, Müller T (2007). Evidence for trans-border movement of rabies by wildlife reservoirs between countries in the Balkan Peninsular. Vet Microbiol.

[12] Johnson N, Freuling C, Vos A, Un H, Valtchovski R, Turcitu M, Dumistrescu F, Vuta V, Velic R, Sandrac V, Aylan O, Müller T, Fooks AR (2008). Epidemiology of Rabies in Southeast Europe. Dev Biol (Basel).

[13] Johnson N, McElhinney LM, Smith J, Lowings P, Fooks AR (2002). Phylogenetic comparison of the genus *Lyssavirus* using distal coding sequences of the glycoprotein and nucleoprotein genes. Arch Virol.

[14] Kissi B, Tordo N, Bourhy H (1995). Genetic polymorphism in the rabies virus nucleoprotein gene. Virology.

[15] Matouch O, Vitasek J, Semerad Z, Malena M (2007). Rabies—free status of the Czech Republic after 15 years of oral vaccination. Rev Sci Tech.

[16] McElhinney LM, Marston D, Johnson N, Black C, Matouch O, Lalosevic D, Stankov S, Must K, Smreczak M, Żmudziński JF, Botvinkin A, Aylan O, Vanek E, Cliquet F, Müller T, Fooks AR (2006). Molecular epidemiology of rabies viruses in Europe. Dev Biol (Basel).

[17] Metlin AE, Cox J, Rybakov SS, Huovilainen A, Grouzdev KN, Neuvonen E (2004). Monoclonal antibody characterization of rabies virus isolates from Russia, Finland and Estonia. J Vet Med B.

[18] Metlin AE, Rybakov SS, Gruzdev KN, Neuvonen E, Cox J, Huovilainen A (2006). Antigenic and molecular characterization of field and vaccine rabies virus strains in the Russian Federation. Dev Biol (Basel).

[19] Müller T, Bätza HJ, Beckert A, Bunzenthal C, Cox JH, Freuling CM, Fooks AR, Frost J, Geue L, Hoeflechner A, Marston D, Neubert L, Revilla-Fernández S, Vanek E, Vos A, Wodak E, Zimmer K, Mettenleiter TC (2009). Analysis of vaccine—virus—associated rabies cases in red foxes (*Vulpes vulpes*) after oral rabies vaccination campaigns in Germany and Austria. Arch Virol.

[20] Orlowska A, Smreczak M, Trebas P, Zmudzinski JF (2008). Comparison of real-time PCR and heminested RT-PCR methods in the detection of rabies virus infection in bats and terrestrial animals. Bull Vet Inst Pulawy.

[21] Picard-Meyer E, Robarded E, Moroz D, Trotsenko Z, Drozhzhe Z, Biarnais M, Solodchuk V, Smreczak M, Cliquet F (2012). Molecular epidemiology of rabies in Ukraine. Arch Virol.

[22] Pötzsch CJ, Kliemt A, Klöss D, Schröder R, Müller W (2006). Rabies in Europe—trends and developments. Dev Biol (Basel).

[23] Rihtaric D, Hostnik P, Grom J, Toplak I (2011). Molecular epidemiology of the rabies virus in Slovenia 1994–2010. Vet Microbiol.

[24] Smreczak M, Orlowska A, Trebas P, Zmudzinski JF (2008). Application of heminested RT-PCR to the detection of EBLV1 and classical rabies virus infections in bats and terrestrial animals. Bull Vet Inst Pulawy.

[25] Tamura K, Dudley J, Nei M, Kumar S (2007). MEGA4: molecular evolutionary genetics analysis (MEGA) software version 4.0. Mol Biol Evol.

[26] Turcitu MA, Barboi G, Vuta V, Mihai I, Boncea D, Dumitrescu F, Codreanu MD, Johnson N, Fooks AR, Müller T, Freuling CM (2010). Molecular epidemiology of rabies virus in Romania provides evidence for a high degree of heterogeneity and virus diversity. Virus Res.

[27] Thoulouze MI, Lafage M, Schachner M, Hartmann U, Cremer H, Lafon M (1998). The neural cell adhesion molecule is a receptor for rabies virus. J Virol.

[28] Vos A (2003). Oral vaccination against rabies and the behavioural ecology of the Red Fox (*Vulpes Vulpes*). J Vet Med.

[29] Vos A, Pommerening E, Neubert L, Kachel S, Neubert A (2002). Safety studies of the oral rabies vaccine SAD B19 in stripped skunk (*Mephitis mephitis*). J Wild Dis.

[30] Wandeler AI (2008). The rabies situation in Western Europe. Dev Biol (Basel).

[31] Zienius D, Zilinskas H, Sajute K, Stankevicius A (2009). Comparative molecular characterization of the rabies virus in the Lithuanian raccoon dog population. Bull Vet Pulawy.

